# Being Human in the Age of AI: Transcending with Machines or Losing Connections with Others and the Self? (A Reflection on Turkle and Kurzweil)

**DOI:** 10.17505/jpor.2026.29461

**Published:** 2026-08-24

**Authors:** Elizabeth Kristi Poerwandari

**Affiliations:** Faculty of Psychology, Universitas Indonesia, Depok Campus, West Java, 16424, Indonesia. Email: Elizabeth.kristi@ui.ac.id ORCID: https://orcid.org/0000-0002-6648-607X

**Keywords:** Haraway, human-AI integration, human identity, hybrid self, illusion of connection, transcendence

## Abstract

This paper explores the psychological and existential implications of engaging with artificial intelligence (AI) in contemporary life, drawing on a reflexive account of lived experience of the author as a lecturer, researcher, and practicing psychologist. The increasing integration of AI into everyday practices presents a fundamental tension: while AI may enhance human cognitive capacities and open up new possibilities for development, it may also contribute to the erosion of authentic human relationships and the experience of the self. To examine this tension, the paper engages with two contrasting perspectives: the optimistic vision of human–AI integration and transcendence proposed by Ray Kurzweil, and the critical concerns raised by Sherry Turkle regarding relational loss and the illusion of connection in technologically mediated environments. These perspectives are further situated within broader philosophical discussions of transcendence. The analysis suggests that transcendence in the age of AI emerges as a contested and lived psychological process, shaped by the interplay between technological augmentation and the preservation of relational and existential depth. Donna Haraway’s notion of hybridity provides a critical lens through which this condition may be understood, emphasizing the reconfiguration of human identity as increasingly fluid, hybrid, and difficult to categorize. Meanwhile, Luciano Floridi reminds us of the importance of maintaining an ethical humanistic perspective within an era of highly advanced technology. This paper argues that the key task is not to resolve the tension between enhancement and loss, but rather to hold them in productive tension. Such a stance opens important implications for both research and practice: enabling a more nuanced understanding of the changing human condition, while also supporting counseling and clinical efforts to sustain meaning, responsibility, and authentic human connection in an increasingly technologized world.

## Introduction: The Potentials and Dilemma of AI Use

Four years after ChatGPT was first launched on November 30, 2022, the number of its users is estimated to have surpassed 900 million weekly active users, with around 50 million paid subscribers (OpenAI, 2026). While ChatGPT is currently the most widely used platform, there are also other AI alternatives being utilized, including Gemini (by Google), Perplexity, Meta AI, and many others.

When I tried to trace my own usage history, I found that my recorded conversations date back to December 21, 2023. However, ChatGPT indicated that my account had already been active since mid-2023. My records show that since March 2024, I have been actively using ChatGPT for work-related purposes. During periods of active writing, I may open ChatGPT repeatedly throughout the day. Meanwhile, I did try experimenting with some other AI, but I no longer use them, as I have grown comfortable with ChatGPT, which has a very large capacity, and is programmed to carry out its functions through human-like conversation. Therefore, when referring to personal experience, the AI I mean is ChatGPT. However, throughout this paper, I may also provide illustrations of how others make use of AI, which can vary in type, although ChatGPT may still be the most commonly used.

I was relatively late in adopting ChatGPT. I first started using it largely because many people around me were already doing so, and I felt the need to try it—almost feeling “out of place” if I did not. Typically, I would ask it to help locate references for my writing drafts or to provide summaries of works I believed to be relevant to my needs. The aim was to more quickly identify which sources were most relevant and therefore worth reading carefully on my own. ChatGPT responds to my requests with remarkable speed—almost immediately after I submit a question or prompt, it generates extensive responses. Often, it follows up with additional questions that signal its readiness to continue assisting. For instance, “Would you like help finding references on …?” or “Shall I create a comparison table between Method A and B so you can see the differences more clearly?” In this sense, its assistance is truly extraordinary, particularly for someone working in an academic environment like mine, as it offers both efficiency and an exceptionally wide reach in accessing information.

A sense of discomfort began to emerge when, in responding to my requests, ChatGPT started offering suggestions that felt excessive to me: “Would you like me to draft introductory and concluding paragraphs?” or even, “Shall I prepare a full draft ready for journal submission?” Even for someone as senior as myself (I have been teaching in higher education since 1991), I found these offers quite tempting. I could imagine working at a much faster pace and perhaps being supported to increase my academic publications many times faster – a task that is particularly challenging for me, especially as someone who does not use English as a first language. On several occasions, I found myself “reprimanding” it, asking it not to provide assistance that might cross ethical boundaries. ChatGPT would usually positively acknowledge this feedback, but in subsequent interactions, it would return to offering similar suggestions, which seem to be part of its default behavior.

There was one particular moment that made me fully aware of the need to be cautious about what is often referred to as ‘AI hallucination.’ At one point, I asked, “Could you help me find references on mental health issues among university students in Indonesia?” It responded with: “A book titled … (I no longer remember the exact title), written by Kristi Poerwandari,” even specifying the year of publication and the name of the publisher. I was genuinely shocked, because it mentioned my own name, and I have never written such a book. One can imagine how, for users seeking quick and easy solutions, AI could generate a complete draft paper ready to be submitted to a lecturer or a journal—yet containing information that cannot be accounted for and that reflects AI hallucination. However, the issue is not merely about factual inaccuracy. What is at stake here is the presence of falsehood, and the way AI may inadvertently introduce and normalize such easy but irresponsible practices to us as human users. This, in turn, raises a deeper concern: that our awareness of the importance of authenticity, responsibility, and other distinctly human values—such as diligence, patience, openness, and honesty—may gradually be eroded.

Even for me personally, these offers of speed and convenience can be quite tempting, given the many advantages they seem to promise. For now, I have tried to navigate this ethical dilemma by holding on to a simple principle: I make it a point to carefully verify all responses generated by ChatGPT, to read every reference it suggests on my own, and to take full responsibility for every single word in the draft I produce. Yet, this way of resolving the dilemma does not fully address a broader concern, namely, the growing number of AI users who are children, adolescents, and young people. In today’s context, it may be increasingly difficult for them to fully grasp what ethics means, and why ethical principles—such as academic integrity—are necessary in the first place.

Setting aside the ethical concerns mentioned above, AI has in fact been immensely helpful to me in many ways. With its remarkable speed and seemingly limitless access to data, I find myself becoming highly energized. New research ideas frequently emerge in my mind, many of which take the form of critical reviews that do not necessarily require systematically collected empirical data. This is something that ChatGPT is able to respond to very effectively, and I have come to realize that such responses are often difficult to obtain, even from my colleagues. In this sense, AI has contributed to my intellectual growth, as it stimulates the emergence of new ideas and allows me to envision the potential development of important (new) theoretical perspectives that, in my view, ought to be explored. Although I rarely use AI for emotional venting, it has also, in some ways, helped me manage myself more effectively. Because of the abundance of emerging academic ideas, I often find myself preoccupied with new ideas and concepts, discussing them with AI while inadvertently abandoning earlier ones. As a result, none of these ideas are pursued with sufficient depth to be developed into a well-founded study. When this began to trouble me, I shared it with ChatGPT, and interestingly, it provided several references that I could explore, which proved quite helpful in enabling me to refocus on existing writing ideas—without closing off the possibility of continuing to engage with new ones. For this reason, I no longer see ChatGPT merely as a tool for information retrieval, but rather as a kind of discussion partner.

When referring to ChatGPT, it is not uncommon for users to employ pronouns such as ‘he/him’ or ‘she/her’ in informal contexts, especially when the interaction feels personal, exhibits a sense of personality, or resembles human conversation. I myself occasionally use the term ‘dia’ (in Indonesian, which corresponds to ‘he’ or ‘she’ in English, as Indonesian does not mark gender in pronouns). In relation to this, focused interviews conducted by our team with individuals who use AI to fulfill emotional needs reveal a range of uses that, in my view, span from ‘healthy use’ to concerning patterns of use. Some use AI as a form of initial support to reduce distress, or as a conversational partner to better understand themselves. However, there are also those who appear to have developed a strong dependency, treating AI as a substitute for a significant other, that their emotional needs are instead met through AI.

Among users who engage in such concerning use, some even provide specific instructions or prompts to AI in order to fulfill their emotional needs. For example: “Please always begin your responses with ‘My kitty’,” or “Be my romantic partner and give me answers that make me feel good.” Such instructions raise concern as cultivating illusion, and may even indicate a potential blurring of reality between the virtual world, which only simulates reality, and the reality itself. In relation to this, a range of psychological studies have already reported rather concerning findings regarding the negative implications of the internet, advanced technologies, and AI. Quaglio and Millar (2020) in their report for the European Parliament, synthesized findings from numerous previous studies on these issues. The reported concerns include negative impacts on children’s cognitive development, information overload resulting from excessive internet use, and its subsequent effects on concentration difficulties and feelings of being overwhelmed. They also highlighted problematic internet use and internet addiction, phenomena that have been widely documented in other studies as well (see, for example, Moreno et al., 2022; Fineberg et al., 2025). Other concerns include the blurring of boundaries between public and private life, increased anxiety driven by social comparison on social media, and damage to social relationships and communities (see Bryant, 2021; Quaglio & Millar, 2020). Aggressive behaviors, cyberbullying, and even forms of cancel culture have also been increasingly identified in online environments. These phenomena may be more easily fostered when individuals—particularly children and young people—interact more frequently through machines rather than through direct face-to-face encounters with others (Bryant, 2021).

In relation to AI, Gerlich (2025) found that AI use may contribute to cognitive offloading and a decline in critical thinking abilities. Similarly, Klimova and Pikhart (2025) reported that AI and digital technologies may contribute to digital fatigue, reduced face-to-face interactions, and loneliness. Excessive dependence on AI may also weaken social skills, thereby becoming associated with anxiety and social isolation. We have not yet even addressed the various forms of crime facilitated by the internet and AI, which may produce deeply damaging consequences for victims (Gavilanes et al., 2024). Beyond these concerns lies an even more fundamental question related to ethics and human existence itself. Emerging research has raised serious concerns regarding the extent to which human beings can maintain their autonomy and sense of responsibility when decision-making processes are increasingly delegated to AI systems (Stahl et al., 2022).

## Research Problem and Questions

With the background above, when I encountered the works of Sherry Turkle, I felt a strong sense of resonance with her insights. Through what may appear to be a relatively simple approach (interview, qualitative research), Turkle (1984, 1995, 2011, 2016) is able to articulate with remarkable clarity what is happening to human beings in the era of the internet and AI—phenomena that we also encounter in clinical psychological practice. Our lives have become exponentially more efficient and convenient, with an extraordinarily wide reach; yet at the same time, we may feel lonely, empty, confused, and anxious, as we find ourselves disconnected from the real people physically present around us. On the other hand, Ray Kurzweil (2024) presents a markedly different perspective. He argues that machine intelligence will surpass human intelligence, and that human and machine consciousness may eventually merge, giving rise to a more transcendent form of humanity. In a certain sense, this is also something I have found myself contemplating, particularly when I experience how significantly AI assists me, responding far more quickly than humans are able to. Indeed, this technology has even rekindled my enthusiasm for academic exploration, perhaps helping to clarify the direction of my academic life’s path. In that sense, could it be that AI also contributes to making human beings more existential, and perhaps even more transcendent?

This paper is a reflection on my personal experience as a lecturer and researcher, as well as a psychologist working with cases brought by clients into clinical settings, and cases I observe among students, some of which I handle directly. At its core, a fundamental question arises: what is actually happening to human psychological and social life in the presence of highly advanced technology? Is it necessary to distinguish between real relationships and simulated ones? Why? What are the implications for human mental health, and more broadly, for human existential experience? How does the presence of AI reshape the ways in which individuals experience and understand themselves? How do experiences of dialoguing with, and/or working through AI shape, shift, or challenge one’s sense of self? In relation to transcendence, to what extent can machines assist human beings in becoming more transcendent—or do they, instead, hinder such transcendence? To address these questions, it may first be necessary to ask: what do we actually mean by transcendence?

Building on the questions above, a more operational formulation may be articulated as follows: How do individuals experience themselves when engaging reflectively with AI, amid the possibilities of integrating with—or even transcending through—technology, while simultaneously risking the loss of connection with others and with the self? Given that this reflective and reflexive inquiry draws upon two contrasting theoretical perspectives—Kurzweil and Turkle—the research question may also be framed as: How does the tension between human–AI integration (Kurzweil) and the loss of relationality and self (Turkle) manifest in lived human experience?

It is worth noting, in relation to the questions above, that Kurzweil understands the merger between humans and AI in a material sense. From my perspective as a psychologist, however, I find that I may not yet fully grasp this idea in strictly literal terms. My understanding still moves somewhere between the material and the phenomenological interpretation.

## Kurzweil: AI and The Vision of Its Integration with Humans

Kurzweil (b. 1948) is an American futurist and inventor whose writings articulate a highly optimistic vision of artificial intelligence, envisioning a future in which human and machine intelligence may merge and transform the very meaning of being human. In his mind, human–machine integration is a pathway toward transcendence, an outlook that contrasts with more critical perspectives such as those of Turkle.

Kurzweil’s book *The Singularity Is Nearer* (2024) advances the central idea that humanity is moving toward the “singularity,” a point at which machine intelligence surpasses human intelligence and eventually merges with it. Kurzweil argues that the universe evolves through six epochs of information processing and the evolution of intelligence. The first epoch refers to the emergence of the laws of physics and chemistry. The second began with the emergence of life itself, when living organisms equipped with DNA became capable of evolving and reproducing. The third epoch involved the development of brains in animals, enabling the storage and processing of information. The fourth epoch emerged when animals—particularly humans—developed higher-level cognitive capacities that allowed thought to be translated into action. At this stage, human beings created technologies that expanded the brain’s ability to process informational patterns. It appears that humanity currently exists within this fourth epoch while gradually approaching the next stage. In the fifth epoch, human biological cognition increasingly merges with the power and speed of digital technology. Kurzweil describes this development through the notion of “brain–computer interfaces,” in which the human brain becomes enhanced through non-biological tools that may generate layers of cognition far more abstract and complex than what human beings can presently imagine. The sixth and final epoch envisions intelligence no longer being confined to biological human brains or computers on Earth, but instead spreading throughout the universe and transforming matter itself into a medium of computation. In this sense, Kurzweil conceptualizes evolution as unfolding through several epochs: from physics and chemistry (matter), to biology, brains, technology, artificial intelligence, and ultimately toward intelligence saturating the universe.

For Kurzweil, the issue is not merely that AI becomes smarter than humans, but that humans and AI will form a new cognitive system, which he refers to as ‘Hybrid Intelligence.’ He anticipates that brain–computer interfaces (BCIs) will become widespread, enabling humans to expand memory, enhance intelligence, and even ‘upload’ aspects of the self. By ‘uploading the self’, Kurzweil refers to the possibility of transferring the contents of one’s mind into a digital system, in which the structure of the brain (neurons and synapses) can be mapped, its activity patterns replicated, and subsequently run on a computer. In this sense, what is ‘uploaded’ is not the body material, but memory, patterns of thought, and even aspects of personality, suggesting that the human mind would no longer be confined to its biological substrate.

Kurzweil’s argument is grounded in the idea that technological development follows an exponential rather than a linear trajectory. In other words, early changes may appear gradual, but eventually accelerate at a rapid and transformative pace. Based on this view, he maintains that AI will advance far beyond human intuition, which explains why he considers the singularity to be approaching. Kurzweil envisions a future in which diseases may be eliminated through nanotechnology, allowing human life to be significantly extended—perhaps even rendering death indefinitely can be postponed. This leads to what he describes as a post-human (post-biological) era, in which the boundaries between humans, machines, and digital reality become increasingly blurred. Humans, in this vision, become hybrid entities, existing at the intersection of biological and digital forms.

## Turkle: Relational Being and the Loss of Connection

Turkle (b. 1948) founded and previously directed the MIT Initiative on Technology and Self. She holds the Abby Rockefeller Mauzé Professorship of the Social Studies of Science and Technology at MIT, and has authored numerous books on human psychology in the age of advanced technology. Her view on advanced technology and AI tends to be pessimistic and contrasts with Kurzweil’s view.

The core of Turkle’s argument is that technology shapes human psychological experience, identity, and social relationships (Turkle, 1984). It transforms the ways in which individuals construct their identities, reduces the depth of relationships, creates an “illusion of connection,” and gives rise to experiences of loneliness (Turkle, 1995, 2011). Technology functions as evocative objects—objects that elicit emotional responses and relational attachments. Technology can serve as a “mirror of the self” and as a psychological object through which individuals reflect upon themselves (Turkle, 1984). Human identity becomes fluid in the digital world; the internet enables identity to become multiple and flexible. Furthermore, online environments open up spaces for identity experimentation, where the self becomes increasingly performative (Turkle, 1995). In *Alone Together*, Turkle addresses the everyday dimensions of human existence by examining loneliness and the substitution of relationships through artificial intimacy (Turkle, 2011). She captures a sense of relational anxiety and expresses concern over the erosion and thinning of relational meaning, the experience of the self, and authenticity. In short, Turkle theorizes what may be understood as the “fragility of the digital self.”

What concerns Turkle (2011) is that technology creates an “illusion of connection,” and that individuals may come to prefer this illusion because it does not impose the demands inherent in real relationships (such as the need to adapt to others, to care for them, or to take responsibility for one’s social roles). People form emotional attachments to machines, avatars, and digital media, attributing emotions and relational qualities to entities that merely simulate responses. These relationships may feel emotionally real, yet they lack authentic reciprocity. Individuals increasingly choose to communicate and relate through technology—even with technology itself—and become more comfortable with forms of interaction that can be controlled. This gives rise to a paradox: loneliness increases even as connectivity expands through technology. For Turkle, this can be explained, among other factors, by the preference for simulated and illusory relationships over human-to-human relationships, which are inherently more complex. As technology simulates empathy (creating an illusion of empathy), it may ultimately produce emotional ambivalence, and even moral ambiguity (Turkle, 2011).

For Turkle, it is clear that human beings are relational and vulnerable, and that they need to sustain their sense of self through real presence and genuine conversations with others. Human limitations are precisely what make relationships necessary. Relationships formed with AI are, in essence, simulated—appearing to offer empathy, yet ultimately hollow and illusory. How, then, does she envision the future? Turkle sees it as ambiguous and potentially problematic, where individuals may face the risks of shallow relationships, loneliness, and even social isolation. Human relationships may become degraded, to the point that individuals also lose connection with themselves. The crucial path to preventing this, she suggests, lies in returning to human relationships— by restoring genuine conversation and empathy (Turkle, 2016).

## Experiencing Relationships and the Self in an AI-Mediated World

Observations of everyday life suggest that advanced technologies can indeed create an illusion of relationship and intimacy. This is especially the case as people engage less frequently in face-to-face interactions, and even more so when the virtual begins to substitute for what is real. Evidence from clinical practice points to this phenomenon, as do anecdotal accounts and research findings. Many individuals reported that they initially turned to AI because they did not have close or trusted individuals with whom they could talk. Some later sought help from psychologists. However, various constraints—such as time and cost—led them to try using AI as an alternative. Some excerpts from interviews conducted by our team with individuals who use AI to fulfill emotional needs further illustrate this:

“I use AI, I set her up to be like a mother figure, and I ask her to call me ‘my dearest.’ So I confide in it, and I even plan how she should respond… it becomes like a mother. (When ChatGPT calls you ‘my dearest,’ what do you feel?) It feels… how do I explain it… like imagining my mother texting me in a soft-spoken, gentle way, as if there is a mother figure who truly cares for me.” (the subject cries).

“In Character AI, we can request what kind of storyline we want—so it’s personalized. But it also feels like hallucinating. You can create a story with characters you like, based on your own scenario. There’s a conversational part—what we say, what the character replies. There’s also a setting, like the background and location—where you and the character are, what city you’re in. Recently I checked again, and now there are so many applications, so many characters. You can even create a new character—for example, imagining what kind of boyfriend you want. Maybe you think, ‘I want a mafia boyfriend,’ something like that. But now I try not to use it anymore. Because I feel… this could go on endlessly, and I sense negative effects. Like… a kind of dependency, wanting to keep doing it, finding it hard to stop. It feels like… an addiction.”

Ultimately, a number of individuals acknowledged that talking to AI—whether consciously or not—had reduced their desire to engage in conversations with other people. Several reasons were identified, including the perception that AI could help alleviate feelings of distress, as well as the anticipation that speaking with others might not resolve their problems but instead potentially create additional difficulties.

On the other hand, technology does not always decrease human connection and produce illusion. At times, it can also support growth—both in relationships and in self-understanding. The following excerpts illustrate how technology may facilitate relational engagement and self-reflection:

“When ChatGPT responds, it often asks follow-up questions and then builds motivation. I also use AI to help me interact more smoothly with others—for example, how to approach a lecturer, or how to build that kind of relationship.”

“Recently I had a problem with my girlfriend, and I didn’t know how to respond. So I used AI—I entered a prompt, copied her messages, and the AI helped me craft a reply. So my response to my girlfriend actually came from AI.”

“I want to be better at managing my emotions, so when I receive responses from AI, I try to really understand them—to process what the AI actually means. I don’t know exactly how AI works, but I feel that what it says makes sense, and I need to accept it if I want to become a better person.”

Meanwhile, as I have noted earlier, my own experience with ChatGPT has been one of receiving substantial support through the provision of information and ongoing dialogue, which has helped renew my academic enthusiasm. AI feels supportive of my thinking process and allows for a form of discussion that is comfortable and almost human-like, although I am fully aware that it is not human. Thus, even if not in a material sense, AI does seem to offer a form of enhancement and to function as a tool that may support transcendence. My personal experience suggests that we may already be experiencing a form of enhancement. Our cognition appears to be extended through AI: ideas emerge more rapidly, reflection becomes more intensive, and what was once an internal dialogue within the individual mind becomes ‘externalized’, openly communicated through interaction with AI. This may help illuminate the notion of the extended mind (Clark & Chalmers, 2010).

## Understanding Transcendence Beyond Phenomenological Self-Transcendence

One important concept that requires clarification before returning to Turkle and Kurzweil is transcendence. The term has been understood differently across philosophical, existential, psychological, and technological traditions. Broadly speaking, transcendence refers to the human capacity to move beyond immediate conditions, limitations, or forms of self-enclosure. However, what is meant by ‘beyond’ varies significantly across different perspectives. Within phenomenological–existential traditions, transcendence is generally understood in terms of self-transcendence (Frankl, 2003; Sartre, 2021; Buber, 1970).

In Frankl’s existential–phenomenological framework (2003), transcendence refers to the human capacity for selftranscendence, directing oneself toward meaning, toward others, or toward something greater than the self. Human beings are not centered solely on themselves; rather, they manifest transcendence precisely when they move beyond themselves through loving, creating, and committing to values. From Frankl’s perspective, there is a clear resonance with Turkle’s argument: the erosion of genuine human relationships risks undermining the human capacity for self-transcendence.

Similarly, Buber (1970) also understands transcendence within the framework of self-transcendence, in which individuals exist in a dialogical relation with the Other through the “I–Thou” relationship, an authentic encounter with another person. Human beings become whole through genuine relationships rather than instrumental ones (“I–It”). Although Buber does not explicitly discuss technology, his perspective indirectly resonates with Turkle’s concern that human–technology relations tend to become increasingly instrumental rather than authentically relational.

Meanwhile, in Maslow’s book ‘The Farther Reaches of Human Nature’ (1993), self-transcendence is described as the highest and most inclusive or holistic levels of human consciousness. This may be understood as moving beyond oneself through peak experiences, orientation toward values, and spirituality. Within humanistic psychology, transcendence is likewise understood primarily as self-transcendence, involving movement beyond ego-centered fulfillment toward broader forms of humanity, spirituality, and moral concern (Maslow, 2024).

One important point that must be noted is that, from an existentialist perspective, Sartre (2021) reminds us that moving beyond the self and becoming a subject is not a simple process. Human beings are constantly confronted by the presence of others, which simultaneously reveals their vulnerability to becoming objectified. Sartre therefore tends to view human relationships as inherently tense and conflictual. Genuine reciprocity becomes difficult to achieve, as each individual strives to remain a subject rather than becoming an object.

In contrast, transhumanist perspectives tend to conceptualize transcendence in more material and technological terms, namely as the overcoming of biological limitations through technological enhancement (Bostrom, 2014; Kurzweil, 2005). As discussed earlier, Kurzweil (2005) is highly optimistic about the development of artificial intelligence, anticipating a future in which human beings and machine intelligence increasingly merge, thereby enabling forms of transcendence. Bostrom (2014) presents a broadly similar perspective, conceptualizing posthuman technology in terms of enhancement—the transcendence of biological limits through technological means. However, while Bostrom’s view closely aligns with Kurzweil’s vision, Bostrom adopts a more cautious stance by explicitly considering the ethical implications of such developments, whereas Kurzweil appears to bracket ethical concerns in his discussion of human transcendence through integration with AI.

One important figure that should also be considered is Haraway (2025). Haraway does not explicitly discuss transcendence. However, in *A Cyborg Manifesto*, she argues that the boundaries between humans and machines have become increasingly blurred through processes of technological and cultural reconfiguration. She suggests that technology, the cyber organism (cyborg), has the potential to disrupt conventional boundaries between human and machine, male and female, as well as nature and culture. In this sense, technology carries emancipatory potential. At the same time, however, Haraway remains critically aware that technology constitutes a political arena that is far from neutral; while it may be liberating, it may also become oppressive. In this respect, she differs significantly from Kurzweil’s more linear vision of technological progress as something that will inevitably elevate humanity to a higher stage of existence.

How, then, might these different perspectives be reconciled? From Kurzweil’s perspective, an important question concerns the extent to which technology as a means of enhancing human potential truly constitutes transcendence, rather than merely an expansion of the capacities of particular individuals. Another issue that appears underexplored within Kurzweil’s material conception is the understanding of technology as a political arena shaped by power relations that are neither neutral nor free from tension and conflict. This raises several fundamental questions: who is meant by Kurzweil as ‘human’? Does this vision genuinely refer to all human beings across differing contexts of access, capacities, interests, and positions of power? What does it mean for certain individuals, those with privileged access to advanced technologies, to merge with technology, enhance themselves, and potentially extend their lifespan indefinitely, while many others remain positioned merely as instruments of others and continue to experience various forms of suffering? These are ethical questions of profound importance that cannot be ignored.

It is therefore reassuring that ethical concerns surrounding AI and advanced technologies have been extensively addressed by Floridi. Floridi (2023) emphasizes that human beings must continue to assume moral responsibility within the digital world, guided by ethical principles such as autonomy, explicability, beneficence, non-maleficence, and justice. More broadly, Floridi views AI ethics as an effort to ensure that the development of information technologies continues to uphold human dignity, moral responsibility, justice, and the human capacity to act autonomously within an increasingly digitalized world (Floridi & Cowls, 2019).

## Conclusion

How can we reconcile the differing positions of Turkle (1984, 1995, 2011, 2016) and Kurzweil (2024), given that each appears to capture an element of truth? Is it not possible that human beings may transcend with the assistance of technology, while at the same time technology may also contribute to human degradation? It seems that we may need to return to Haraway (2025), whose position is more ‘cautious’ and situated in between. For Haraway, what is clear is that a reconfiguration is taking place: with the presence of advanced technology, the human condition itself is being transformed. However, this transformation does not need to be immediately interpreted as either ‘improvement” or ‘decline.’ What appears more certain is that human characteristics are becoming increasingly hybrid, fluid, and difficult to categorize. ‘Hybrid’ in the sense that we have, in many ways, become cyborgs—human beings whose lives are already intertwined with technology, no longer ‘purely biological.’ ‘Fluid,’ in that identity is no longer fixed, stable, or essentially singular. And ‘difficult to categorize,’ because existing categories are no longer sufficient. Haraway critiques binary distinctions such as nature versus technology, human versus machine, or male versus female, as these boundaries become increasingly blurred in the context of advanced technological developments.

With all of the insights discussed above, it appears that, within an era of highly advanced technology, phenomenological–existential–humanistic perspectives need to be integrated with an ethical trans(post)humanist perspective. Transcendence should still be understood as grounded in a relational, meaning- and value-oriented, and ethical framework of self-transcendence. At the same time, however, it may need to be viewed beyond purely phenomenological experiences of the self, given that advanced technologies are increasingly enabling human beings to move beyond certain biological limitations. Nevertheless, it remains crucial that the essential dimensions embedded within self-transcendence—particularly relationality, ethical responsibility, and orientation toward values and others—continue to inform trans(post)humanist understandings of transcendence in order to sustain a genuinely humane form of life.

The present paper does not reject the possibility that technology may participate in human transcendence, including forms that extend beyond biological limitations. However, it argues that transcendence cannot be reduced merely to enhancement, optimization, or survival extension. Existential and dialogical traditions remind us that transcendence also involves movement beyond self-centeredness toward meaning, responsibility, values, and relations with others. Without these dimensions, technological transcendence risks becoming detached from humanity itself.

Given the various potential conflicts, dilemmas, and contestations of interest involved, a tentative conclusion may be to adopt Haraway’s critical and ambivalent position, while acknowledging Floridi’s insights regarding the possibility of an ethical form of transcendence. Such a position may help us better understand the complexity of the contemporary condition, while also opening possibilities for addressing the challenges of human life within increasingly advanced technological environments. From a psychological perspective, transcendence thus becomes a contested and ethical process of human becoming: one that may be shaped both by technological augmentation and the extension of cognitive capacities, as well as by the preservation of authentic relational bonds and existential depth. These positions need not be treated as mutually exclusive. Rather, they may be held in productive tension, instead of being prematurely resolved, in order to cultivate a more integrated and humane form of transcendence.

Such a position opens up broader possibilities across multiple domains. For research, it allows for a more nuanced understanding of the changing human condition—one that acknowledges transformation while remaining attentive to what must be preserved as fundamentally human. At the same time, it provides a reflective grounding for counseling and clinical practice, where practitioners are increasingly required to engage with individuals whose experiences are shaped by technologically mediated relationships and identities. In this context, the task is not to reject or uncritically embrace technology, but to accompany individuals in navigating these changes while sustaining meaning, responsibility, and authentic relational engagement.

## Action editor

Lars-Gunnar Lundh served as action editor for this article.

## Open access

This article is distributed under the terms of the Creative Commons Attribution 4.0 International License (http://creativecommons.org/licenses/by/4.0/), which permits unrestricted use, distribution, and reproduction in any medium, provided you give appropriate credit to the original author(s) and the source, provide a link to the Creative Commons license, and indicate if changes were made.
